# Supplementary material for: AKT3 Expression in Mesenchymal Colorectal Cancer Cells Drives Growth and Is Associated with Epithelial-Mesenchymal Transition
Source: Cancers (Basel). 2021 Feb 14;13(4):801. doi: 10.3390/cancers13040801 (PMC7918753; doi:10.3390/cancers13040801)
Supplement: Supplementary file 1 [file cancers-13-00801-s001.zip › Buikhuisen, Gomez Barila at al - Cancers - Material S1 - western blot full membrane images.pptx]

## Slide 1
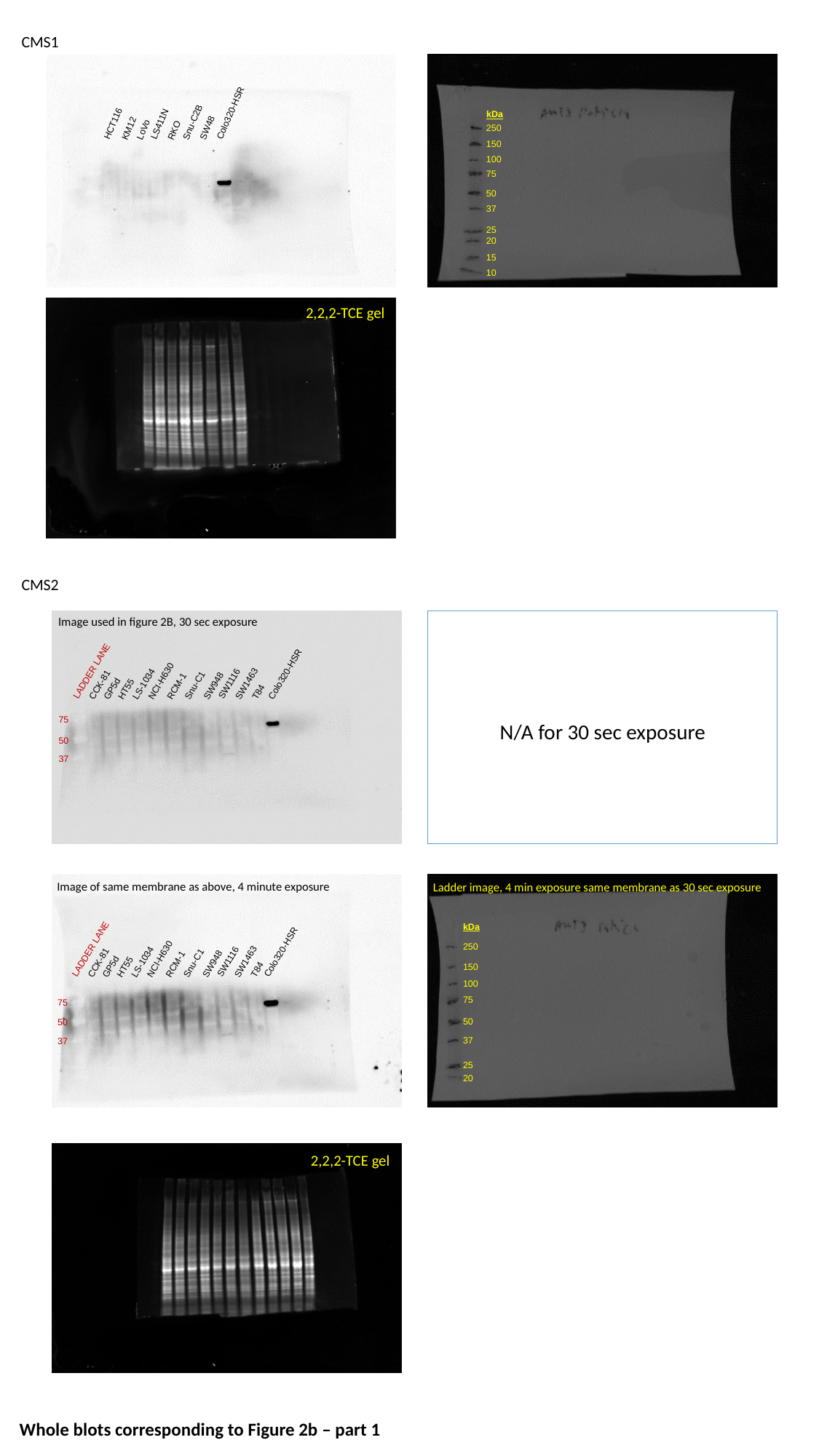

CMS1
Colo320-HSR
kDa
Snu-C2B
HCT116
LS411N
250
SW48
KM12
LoVo
RKO
150
100
75
50
37
25
20
15
10
2,2,2-TCE gel
CMS2
Image used in figure 2B, 30 sec exposure
N/A for 30 sec exposure
LADDER LANE
Colo320-HSR
NCI-H630
SW1116
SW1463
LS-1034
CCK-81
Snu-C1
SW948
RCM-1
GP5d
HT55
T84
75
50
37
Image of same membrane as above, 4 minute exposure
Ladder image, 4 min exposure same membrane as 30 sec exposure
kDa
250
LADDER LANE
Colo320-HSR
NCI-H630
SW1116
SW1463
LS-1034
CCK-81
Snu-C1
SW948
RCM-1
GP5d
150
HT55
T84
100
75
75
50
50
37
37
25
20
2,2,2-TCE gel
Whole blots corresponding to Figure 2b – part 1

## Slide 2
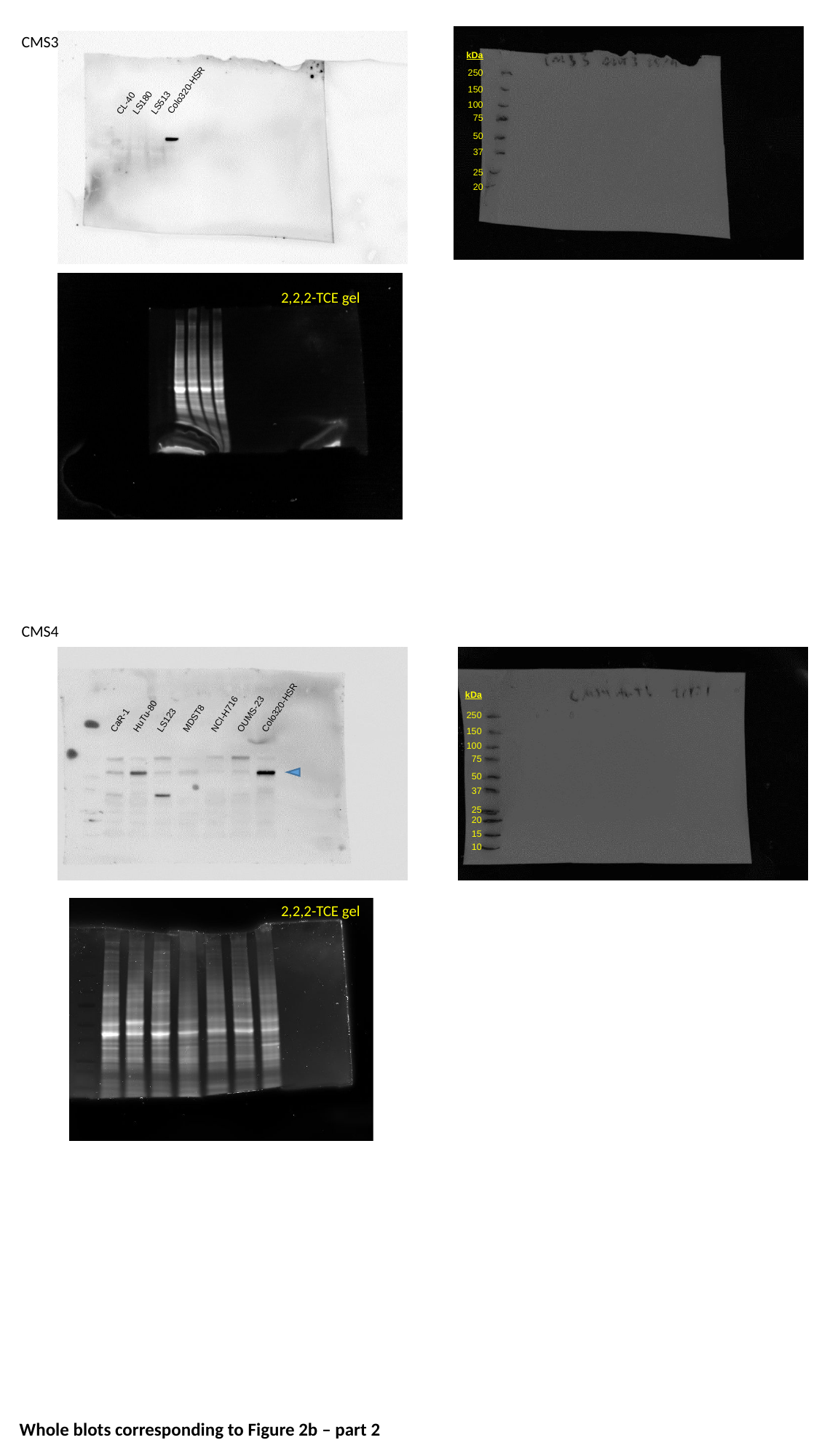

CMS3
kDa
250
150
Colo320-HSR
LS180
LS513
CL-40
100
75
50
37
25
20
2,2,2-TCE gel
CMS4
kDa
250
150
100
75
50
37
25
20
15
10
Colo320-HSR
NCI-H716
OUMS-23
HuTu-80
MDST8
CaR-1
LS123
2,2,2-TCE gel
Whole blots corresponding to Figure 2b – part 2

## Slide 3
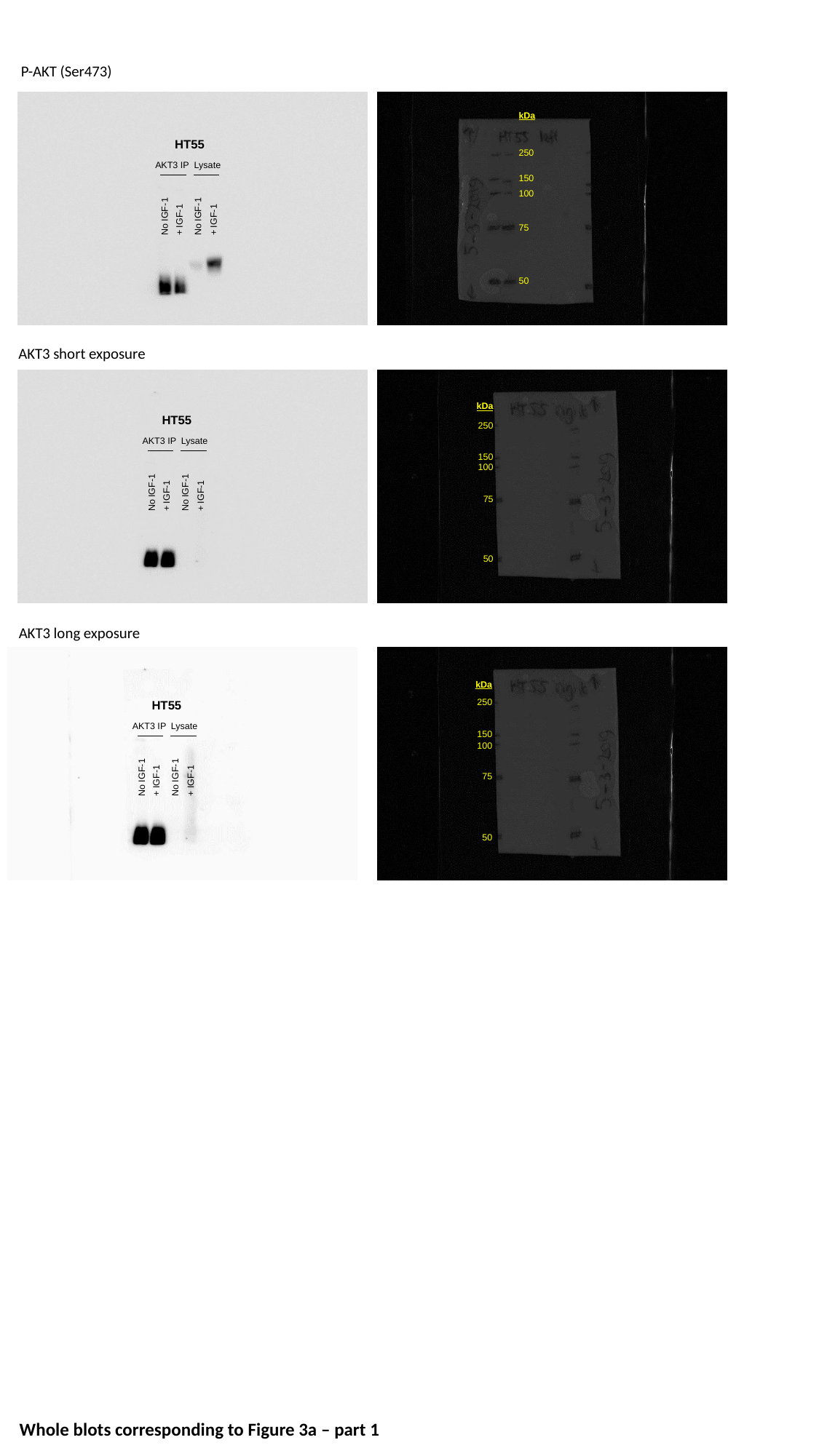

P-AKT (Ser473)
kDa
HT55
250
AKT3 IP
Lysate
150
100
No IGF-1
+ IGF-1
No IGF-1
+ IGF-1
75
50
AKT3 short exposure
kDa
HT55
250
AKT3 IP
Lysate
150
100
No IGF-1
+ IGF-1
No IGF-1
+ IGF-1
75
50
AKT3 long exposure
kDa
250
HT55
AKT3 IP
Lysate
150
100
No IGF-1
+ IGF-1
No IGF-1
+ IGF-1
75
50
Whole blots corresponding to Figure 3a – part 1

## Slide 4
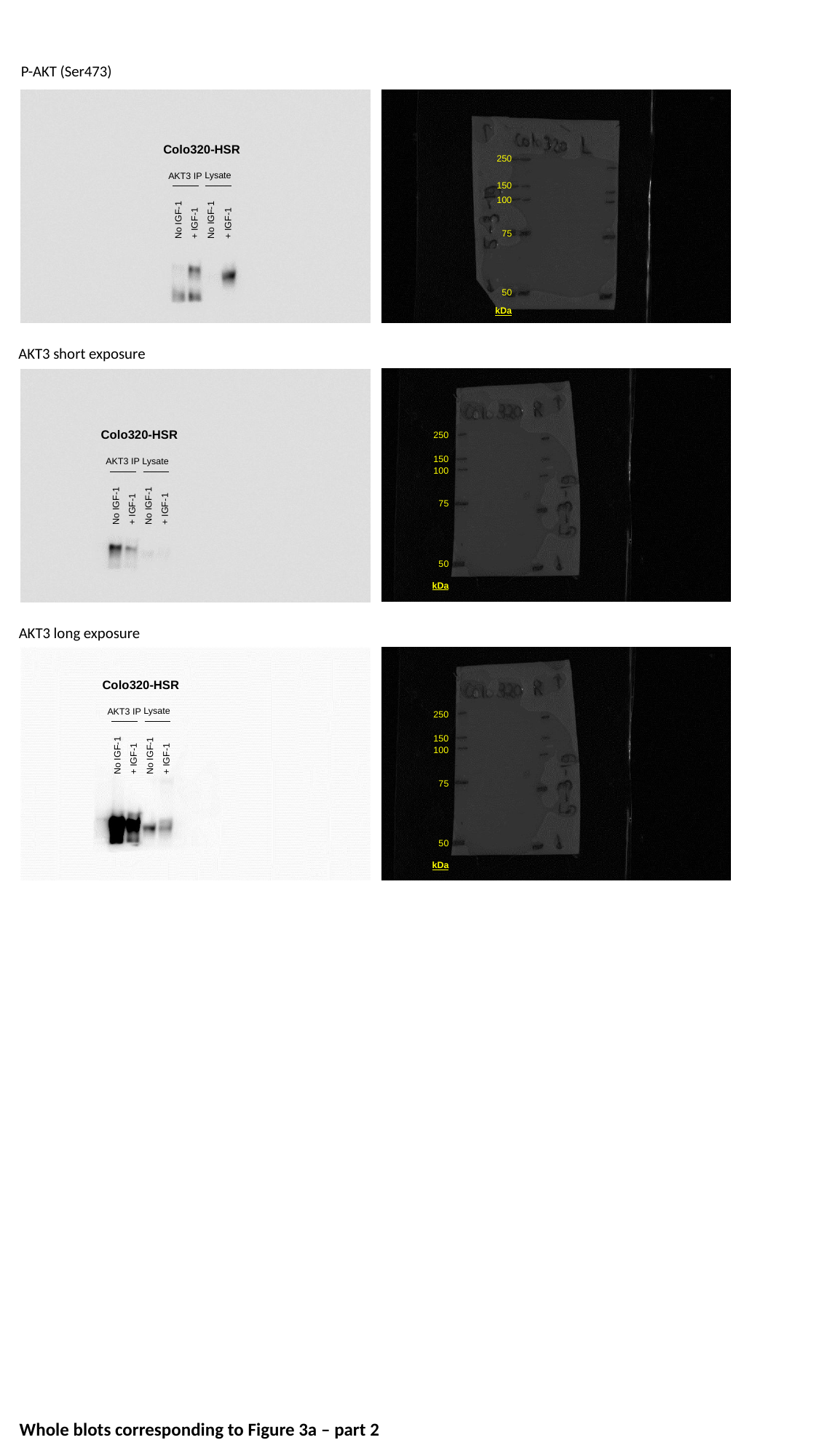

P-AKT (Ser473)
Colo320-HSR
250
Lysate
AKT3 IP
150
100
No IGF-1
No IGF-1
+ IGF-1
+ IGF-1
75
50
kDa
AKT3 short exposure
Colo320-HSR
250
150
Lysate
AKT3 IP
100
No IGF-1
No IGF-1
+ IGF-1
+ IGF-1
75
50
kDa
AKT3 long exposure
Colo320-HSR
Lysate
250
AKT3 IP
150
100
No IGF-1
No IGF-1
+ IGF-1
+ IGF-1
75
50
kDa
Whole blots corresponding to Figure 3a – part 2

## Slide 5
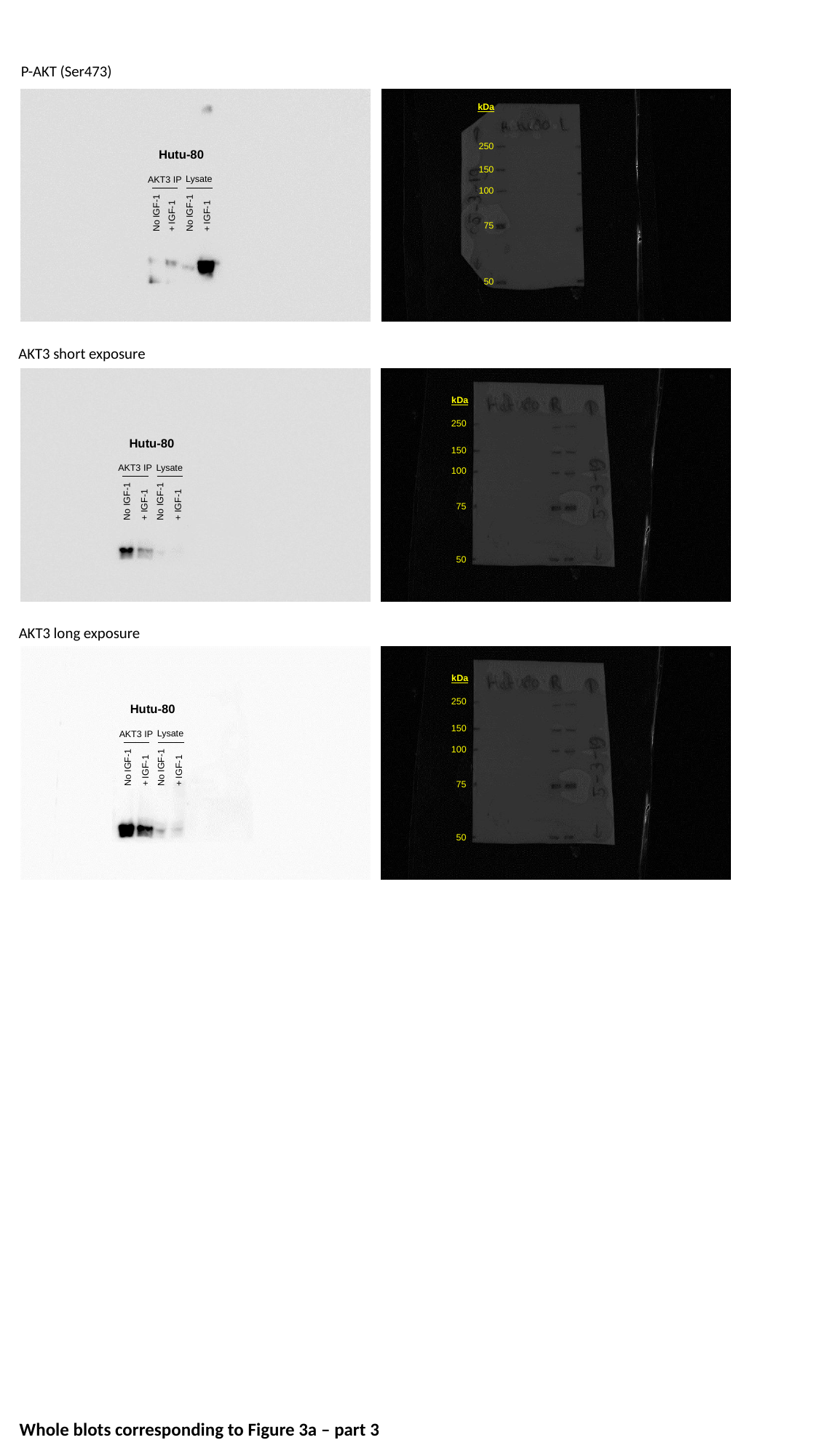

P-AKT (Ser473)
kDa
250
Hutu-80
150
Lysate
AKT3 IP
100
No IGF-1
+ IGF-1
No IGF-1
+ IGF-1
75
50
AKT3 short exposure
kDa
250
Hutu-80
150
Lysate
100
AKT3 IP
No IGF-1
+ IGF-1
No IGF-1
+ IGF-1
75
50
AKT3 long exposure
kDa
250
Hutu-80
150
Lysate
AKT3 IP
100
No IGF-1
+ IGF-1
No IGF-1
+ IGF-1
75
50
Whole blots corresponding to Figure 3a – part 3

## Slide 6
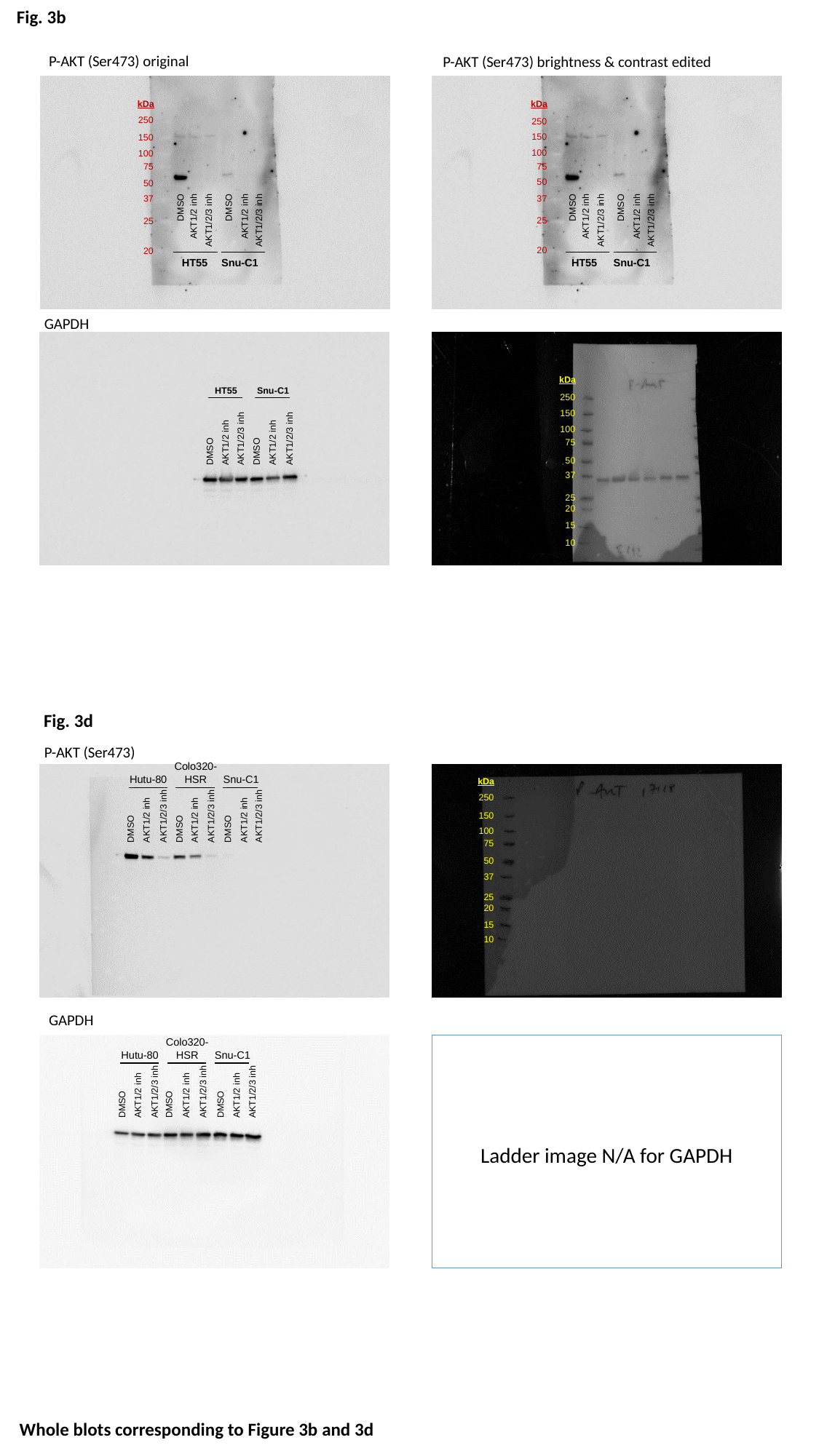

Fig. 3b
P-AKT (Ser473) original
P-AKT (Ser473) brightness & contrast edited
kDa
kDa
250
250
150
150
100
100
75
75
50
50
37
37
DMSO
DMSO
AKT1/2 inh
AKT1/2/3 inh
AKT1/2 inh
AKT1/2/3 inh
Snu-C1
DMSO
DMSO
25
25
AKT1/2 inh
AKT1/2/3 inh
AKT1/2 inh
AKT1/2/3 inh
20
20
HT55
HT55
Snu-C1
GAPDH
kDa
HT55
Snu-C1
250
150
100
AKT1/2/3 inh
AKT1/2/3 inh
AKT1/2 inh
AKT1/2 inh
75
DMSO
DMSO
50
37
25
20
15
10
Fig. 3d
P-AKT (Ser473)
Colo320-HSR
Hutu-80
Snu-C1
kDa
250
AKT1/2/3 inh
AKT1/2 inh
AKT1/2/3 inh
AKT1/2/3 inh
150
AKT1/2 inh
AKT1/2 inh
DMSO
DMSO
DMSO
100
75
50
37
25
20
15
10
GAPDH
Colo320-HSR
Ladder image N/A for GAPDH
Hutu-80
Snu-C1
AKT1/2/3 inh
AKT1/2 inh
AKT1/2/3 inh
AKT1/2/3 inh
AKT1/2 inh
AKT1/2 inh
DMSO
DMSO
DMSO
Whole blots corresponding to Figure 3b and 3d

## Slide 7
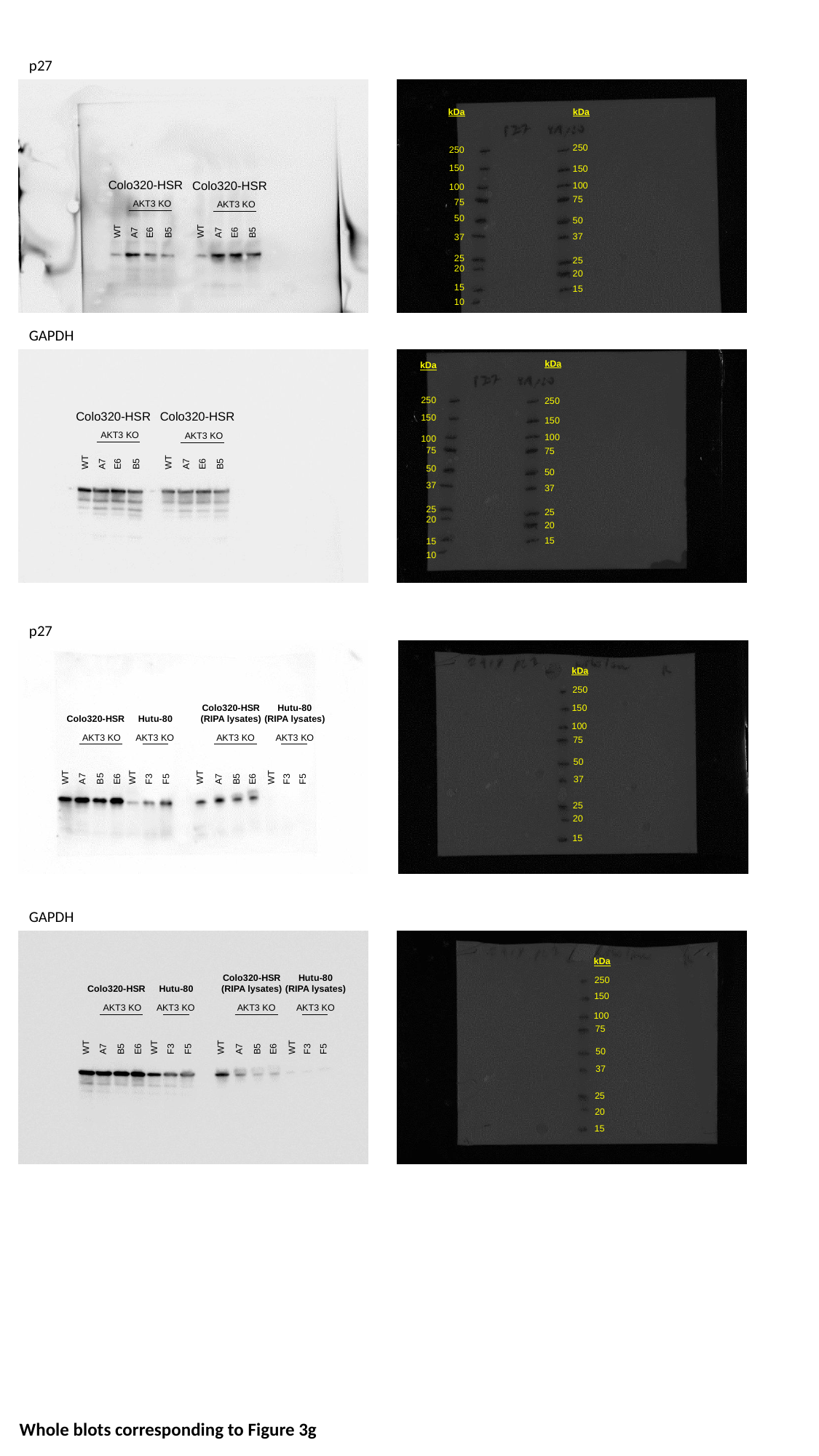

p27
kDa
kDa
250
250
150
150
Colo320-HSR
Colo320-HSR
100
100
75
75
AKT3 KO
AKT3 KO
A7
E6
A7
E6
50
50
WT
B5
WT
B5
37
37
25
25
20
20
15
15
10
GAPDH
kDa
kDa
250
250
Colo320-HSR
Colo320-HSR
150
150
AKT3 KO
AKT3 KO
100
100
A7
E6
A7
E6
75
75
WT
B5
WT
B5
50
50
37
37
25
25
20
20
15
15
10
p27
kDa
250
Colo320-HSR (RIPA lysates)
Hutu-80
(RIPA lysates)
150
Colo320-HSR
Hutu-80
100
AKT3 KO
AKT3 KO
AKT3 KO
AKT3 KO
75
A7
B5
A7
B5
50
WT
E6
WT
WT
E6
WT
F5
F5
F3
F3
37
25
20
15
GAPDH
kDa
Colo320-HSR (RIPA lysates)
Hutu-80
(RIPA lysates)
250
Colo320-HSR
Hutu-80
150
AKT3 KO
AKT3 KO
AKT3 KO
AKT3 KO
100
75
A7
B5
A7
B5
WT
E6
WT
WT
E6
WT
F5
F5
F3
F3
50
37
25
20
15
Whole blots corresponding to Figure 3g

## Slide 8
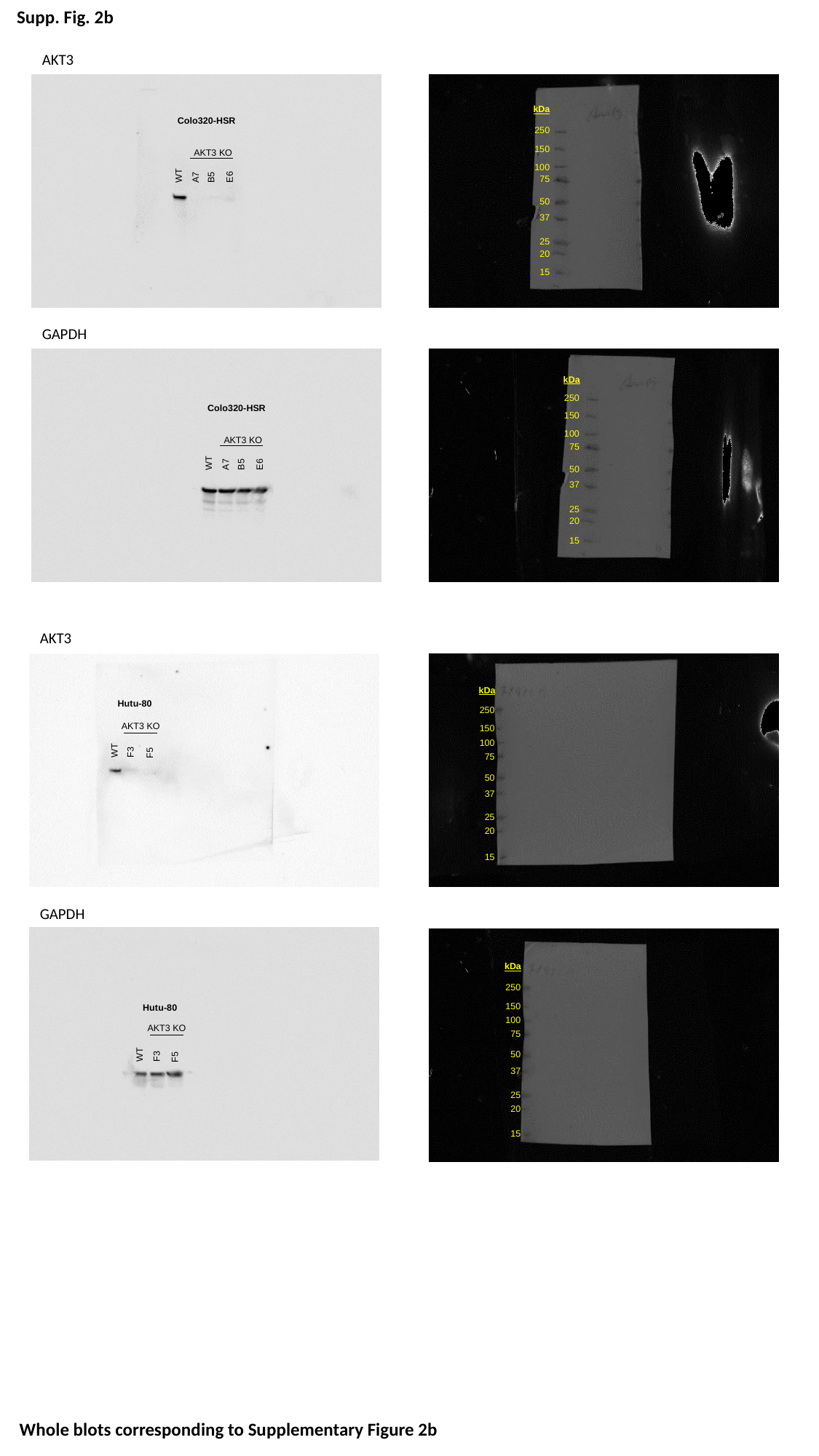

Supp. Fig. 2b
AKT3
kDa
Colo320-HSR
250
150
AKT3 KO
A7
B5
100
WT
E6
75
50
37
25
20
15
GAPDH
kDa
250
Colo320-HSR
150
100
AKT3 KO
A7
B5
75
WT
E6
50
37
25
20
15
AKT3
kDa
Hutu-80
250
AKT3 KO
150
100
WT
F3
F5
75
50
37
25
20
15
GAPDH
kDa
250
150
Hutu-80
100
AKT3 KO
75
WT
50
F3
F5
37
25
20
15
Whole blots corresponding to Supplementary Figure 2b
