# Supplementary material for: AKT3 Expression in Mesenchymal Colorectal Cancer Cells Drives Growth and Is Associated with Epithelial-Mesenchymal Transition
Source: Cancers (Basel). 2021 Feb 14;13(4):801. doi: 10.3390/cancers13040801 (PMC7918753; doi:10.3390/cancers13040801)
Supplement: Supplementary file 1 [file cancers-13-00801-s001.zip › Buikhuisen, Gomez Barila et al - Cancers - Figure S1.pdf]

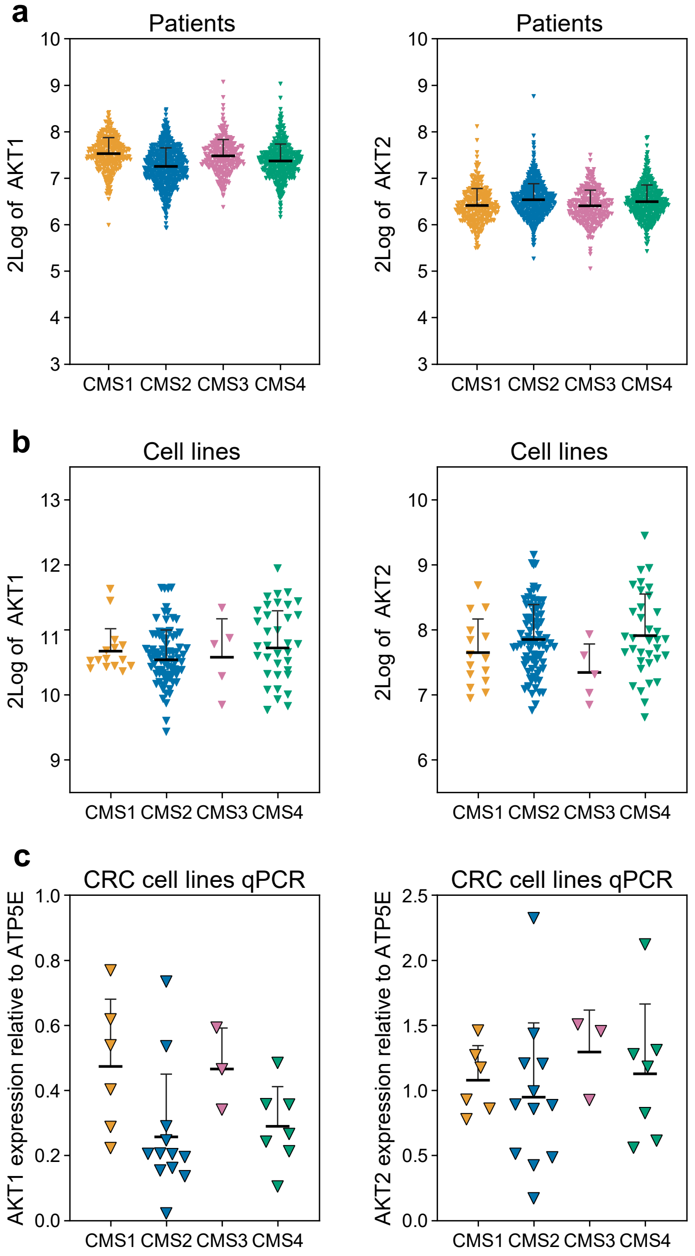

Supplementary Figure 1. AKT1 and AKT2 are not differentially expressed between CMS. (**a**,**b**) 2Log mRNA expression levels of AKT1 and AKT2 in CRC patients (a) and (primary) cell lines (b) as determined by microarray or RNAseq; (**c**) Quantitative real time PCR analysis of AKT1 and AKT2 expression in 28 established CRC cell lines. Expression level was normalised to ATP5E expression, average normalised expression of N=3, each performed with technical triplicates, is plotted per cell line. Normalisation to GUSB yielded similar results.
