# Supplementary material for: AKT3 Expression in Mesenchymal Colorectal Cancer Cells Drives Growth and Is Associated with Epithelial-Mesenchymal Transition
Source: Cancers (Basel). 2021 Feb 14;13(4):801. doi: 10.3390/cancers13040801 (PMC7918753; doi:10.3390/cancers13040801)
Supplement: Supplementary file 1 [file cancers-13-00801-s001.zip › Buikhuisen, Gomez Barila et al - Cancers - Figure S2.pdf]

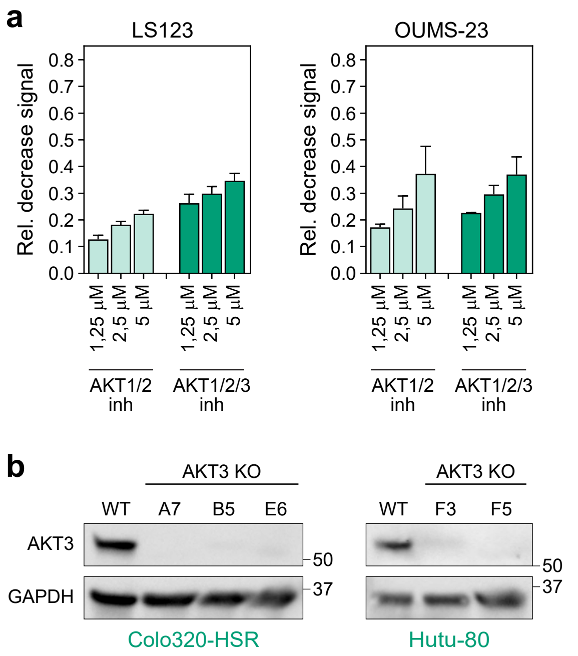

Supplementary Figure 2. CMS4 cell lines lacking AKT3 expression. (**a**) Bar graphs depicting the average decrease in CellTiter Blue signal of low AKT3 expressing CMS4 cell lines upon treatment with AKT1/2 kinase inhibitor and AKT1/2/3 inhibitor (MK2206). All values were normalised to signal in DMSO treated control conditions. Bar graphs indicate mean of three independent experiments, error bars indicate S.D.; (**b**) Western blot images confirming successful CRISPR-based knockout (KO) of AKT3 in single cell clones generated for Colo320-HSR and Hutu-80. WT; Wildtype.
