# Supplementary material for: AKT3 Expression in Mesenchymal Colorectal Cancer Cells Drives Growth and Is Associated with Epithelial-Mesenchymal Transition
Source: Cancers (Basel). 2021 Feb 14;13(4):801. doi: 10.3390/cancers13040801 (PMC7918753; doi:10.3390/cancers13040801)
Supplement: Supplementary file 1 [file cancers-13-00801-s001.zip › Buikhuisen, Gomez Barila et al - Cancers - Figure S3.pdf]

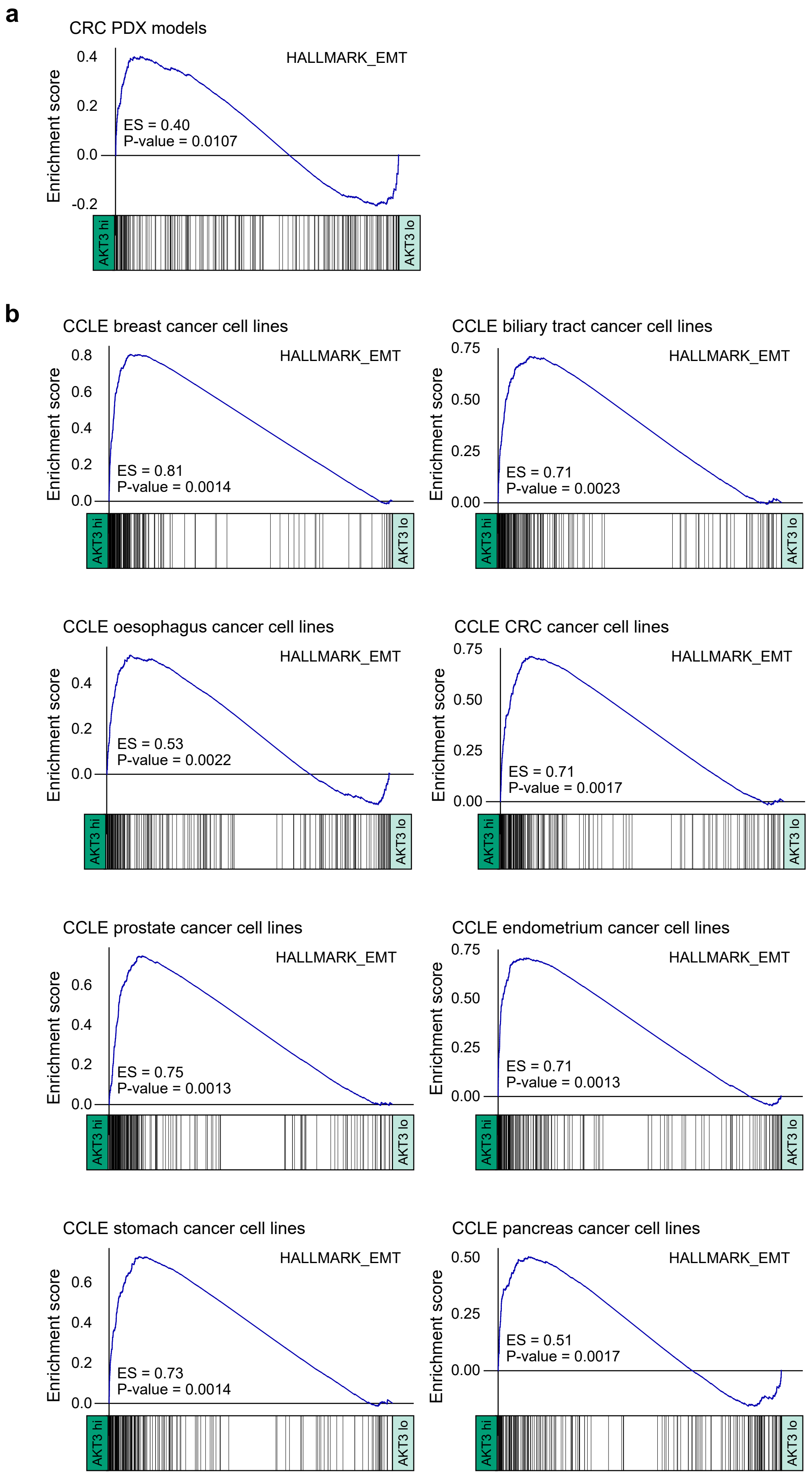

Supplementary Figure 3. EMT gene expression is enriched in pre-clinical models highly expressing AKT3 across multiple carcinoma cell lines. (a) GSEA on PDX models. Cut-off for AKT3 high and low expression was set to 2Log expression >3.5 and <1, respectively; (b) GSEA on 50% high versus 50% low AKT3 expressing carcinoma cell lines included in the CCLE panel, separated per cancer type.
