# Supplementary material for: AKT3 Expression in Mesenchymal Colorectal Cancer Cells Drives Growth and Is Associated with Epithelial-Mesenchymal Transition
Source: Cancers (Basel). 2021 Feb 14;13(4):801. doi: 10.3390/cancers13040801 (PMC7918753; doi:10.3390/cancers13040801)
Supplement: Supplementary file 1 [file cancers-13-00801-s001.zip › Buikhuisen, Gomez Barila et al - Cancers - Figure S4.pdf]

a

Gene	R value	P value
HEG1	0,732	$1,46 \cdot 10^{-10}$
DYNC2H1	0,708	$1,07 \cdot 10^{-9}$
ARMCX2	0,707	$1,14 \cdot 10^{-9}$
LOC100506844	0,707	$1,17 \cdot 10^{-9}$
VIM	0,703	$1,53 \cdot 10^{-9}$
FST	0,695	$2,82 \cdot 10^{-9}$
MLLT11	0,693	$3,22 \cdot 10^{-9}$
ARSB	0,684	$6,14 \cdot 10^{-9}$
TUBA1A	0,675	$1,14 \cdot 10^{-8}$
RBMS3	0,673	$1,31 \cdot 10^{-8}$
CNPY4	0,671	$1,56 \cdot 10^{-8}$
SV2A	0,670	$1,59 \cdot 10^{-8}$
MSRB3	0,670	$1,60 \cdot 10^{-8}$
MAP1B	0,665	$2,29 \cdot 10^{-8}$
FRMD6	0,661	$2,99 \cdot 10^{-8}$

b

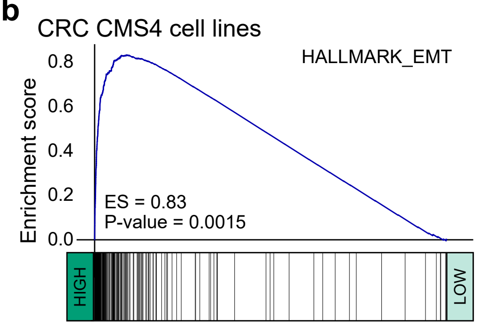

c

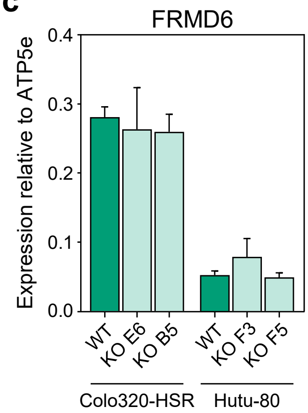

Supplementary Figure 4. FRMD6 expression is correlated to AKT3 expression and EMT in CRC cell lines. (a) Table summarising top 15 genes of which expression positively correlates with AKT3 expression; (b) GSEA on 20% high versus 20% low FRMD6 expressing CMS4 CRC cell lines; (c) Quantitative real time PCR analysis of FRMD6 expression in wildtype and AKT3 KO cell lines. Expression level was normalised to ATP5E expression, average and S.D. of 3 technical replicates is plotted per cell line. Normalisation to GUSB yielded similar results.
